# The English National Lynch Syndrome transformation project: an NHS Genomic Medicine Service Alliance (GMSA) programme

**DOI:** 10.1136/bmjonc-2023-000124

**Published:** 2023-10-30

**Authors:** Kevin J Monahan, Neil Ryan, Laura Monje-Garcia, Ruth Armstrong, David N Church, Jackie Cook, Alaa Elghobashy, Fiona Lalloo, Sally Lane, Frank D McDermott, Tracie Miles, Steven A Hardy, Adele Tyson, Valerie Ya Wen Wang, Anna Kim, Simone Gelinas, Francesca Faravelli, Frances Elmslie, Adam C Shaw

**Affiliations:** 1 Centre for Familial Intestinal Cancer, St Mark's Hospital and Academic Institute, London, UK; 2 Surgery and Cancer, Imperial College London, London, UK; 3 North Thames Genomic Medical Service, London, UK; 4 South West Genomic Medicine Service Alliance, Bristol, UK; 5 Gynae-oncology, University of Edinburgh, Edinburgh, UK; 6 Clinical Genetics, Addenbrooke's Hospital, Cambridge, UK; 7 East of England Genomic Medicine Service Alliance, Cambridge, UK; 8 Wellcome Centre for Human Genetics, University of Oxford, Oxford, UK; 9 Central and South Genomic Medicine Service Alliance, Oxford, UK; 10 Clinical Genetics Service, Sheffield Children's NHS Foundation Trust, Sheffield, UK; 11 Yorkshire and North East Genomic Medicine Service Alliance, Sheffield, UK; 12 Gynae-Oncology, Central and South Genomic Medicine Service Alliance, Wolverhampton, UK; 13 Clinical Genetics, Manchester Centre for Genomic Medicine, Manchester, UK; 14 North West Genomic Medicine Service Alliance, Manchester, UK; 15 Histopathology, Yorkshire and North East Genomic Medicine Service Alliance, Leeds, UK; 16 Colorectal Surgery, South West Genomic Medicine Service Alliance, Exeter, UK; 17 Gynae-Oncology, South West Genomic Medicine Service Alliance, Bath, UK; 18 National Disease Registration Service, NHS England, Newcastle, UK; 19 National Disease Registration Service, South East Genomic Medicine Service Alliance, London, UK; 20 NHS England, London, UK; 21 Clinical Genetics, South East Genomic Medicine Service Alliance, London, UK; 22 National Disease Registration Service, NHS England, London, UK; 23 Clinical Genetics, Guy's and St Thomas' NHS Foundation Trust, London, UK; 24 South East Genomic Medicine Service Alliance, London, UK; 25 Clinical Genetics, North Thames Genomic Medicine Service Alliance, London, UK; 26 Clinical Genetics, St George's Hospital, London, UK

**Keywords:** Colorectal cancer, Endometrial cancer, Genetic markers

## Abstract

**Objective:**

In England, through the Genomic Medicine Service Alliances (GMSAs), a national transformation project aims to embed robust pathways to deliver universal Lynch syndrome (LS) testing for patients with colorectal and endometrial cancers. Prior to commencement of the project, there was evidence of variation and low testing levels in eligible patients which is consistent with other health systems; however, we believe this is amenable to systematic improvement with responsibility for testing delivery by local cancer teams supported by regional infrastructure.

**Methods and analysis:**

A project team and national oversight group was formed in May 2021 with membership including 21×cancer alliances, 7×GMSAs, charities and other stakeholders who agreed key performance indicators. ‘LS champions’ within each cancer team were identified and surveyed. Workforce training focused on effective identification of eligible patients, overcoming barriers and mainstreamed constitutional genetic testing. Comprehensive pathway data analysis was performed in conjunction with the National Disease Registration Service.

**Results:**

Survey and baseline testing data illustrated variation, and a disparity between practice and perception, in levels of testing. The main reported barriers related to funding streams and systematic approaches. Multifaceted training programmes were produced to support workforce development. Champions responsible for testing delivery were appointed in >95% of cancer teams. We identified >9000 historically diagnosed LS patients to support ascertainment for a nationally coordinated screening programme.

**Conclusion:**

This ongoing transformational project is strongly supported by stakeholders in England. Significant quality improvement has been implemented, facilitating systematic delivery of universal testing for LS nationally and reduction in variation in care.

**WHAT IS ALREADY KNOWN ON THIS TOPIC**

- Lynch syndrome (LS) is a cancer susceptibility Mendelian syndrome, which accounts for 1:30 colorectal or endometrial cancers, as well as other cancers.
- There are a range of methods to reduce the lifetime risk of cancer in known carriers, as well as to personalise cancer treatment and improve outcomes.
- LS is a common disease which affects between 1:279 and 400 of the population, therefore, conservatively there are estimated to be 175–200 000 people with this condition in the UK, of whom only 5% have been diagnosed.

**WHAT THIS STUDY ADDS**

- We established the LS transformation project in England, multifaceted national diagnostic programme to deliver diagnosis of LS following a diagnosis of cancer.
- We appointed LS champions in >95% of colorectal and gynaeoncology cancer teams in England, who are responsible for local delivery of testing.
- We provided training and an infrastructure to support delivery of testing, and surveyed the champions to identify areas where support could be provided.
- Performance analysis has been provided in coordination with the National Disease Registration Service, which identifies local and regional variation in practice.

**HOW THIS STUDY MIGHT AFFECT RESEARCH, PRACTICE OR POLICY**

- A national registry has been produced of all historically diagnosed patients to support ascertainment for a new coordinated national colonoscopic screening programme which launched in July 2023.
- The longer-term impact of this work will be evaluated until 2026 when complete data will be made available to measure the impact of this project.
- Regional expert networks have been established to support mainstream genetic testing by local cancer teams, and help manage multidisciplinary care for complex clinical cases.

## Background

Lynch syndrome (LS) is an autosomally dominant inherited condition caused by pathogenic variants in DNA mismatch repair (MMR) genes *MLH1*, *MSH2*, *MSH6* and *PMS2*, or by deletions in *EPCAM* which modifies the expression of *MSH2*.^1^ LS causes increased susceptibility to multiple cancers including colorectal, endometrial and other predominantly epithelial cancers. Cancer incidence in affected individuals varies significantly due to genotype, polygenic modifiers and environmental risk factors.^2–4^

Much of the cancer risk may, however, be mitigated by a range of interventions with a significant impact on life expectancy.^5–7^ These include chemoprevention with aspirin, adenoma surveillance and removal with colonoscopy and risk-reducing surgery.^5 8–10^ In addition, in people with LS who are diagnosed with cancer, there are opportunities for a personalised approach to cancer treatment, which together have significant impacts on cancer-related survival.^1 11–13^

LS affects approximately 1 in 279–400 individuals^14–16^ and, therefore, affects approximately 200 000 people in the UK. Although a common condition, it is estimated that only 5% of patients with LS are known in the UK, thus we miss many opportunities for cancer prevention and improved outcomes following a cancer diagnosis in people with LS. A lack of clear clinical responsibility may be the key reason for underdiagnosis of LS and has been described in the literature as a ‘diffusion of responsibility’.^17 18^

Therefore, there is a clinical imperative to maximise LS diagnosis to ensure those affected are enrolled in effective cancer mitigation programmes and their lifelong care needs are coordinated.

### Tumour testing and the diagnosis of LS

There is consistent evidence of the cost-effectiveness and clinical benefit of a structured diagnostic pathway in patients with LS following a diagnosis of cancer linked to cascade testing in families.^19–25^ In England, the National Institute of Health and Care Excellence (NICE) has developed national guidelines for the National Health Service (NHS) which recommend universal testing for LS in people with colorectal (CRC, DG27)^26^ and endometrial cancer (EC, DG42).^27^ Across England, there are approximately 36 000 CRC and 8000 EC new diagnoses annually and care is managed by over 300 different multidisciplinary cancer teams, who are in turn coordinated by 21 cancer alliances (CAs).

There are three somatic (tumour-based) investigations that can be used for the detection of MMR deficiency (dMMR). Immunohistochemistry (IHC) testing identifies abnormally formed MMR proteins and can indicate the underlying gene in which there is a disease-causing variant. Alternatively, microsatellite instability (MSI), a PCR-based test, identifies errors in repetitive DNA segments called microsatellites which, in the absence of a functional MMR system, are susceptible to insertion–deletion mutations in tumours. Tumour IHC or MSI testing may be performed as an index test in patients with CRC to identify those who may benefit from further assessment for LS^26^ (only IHC to test for dMMR EC^27^). If a tumour is dMMR with either abnormal IHC, or MSI, and no evidence of *MLH1* promoter methylation (in CRC *BRAF* testing is a proxy marker for methylation), the patient is eligible for genetic testing for LS.

### The ‘unmet need’: improving delivery of diagnosis of LS after cancer

Unfortunately uptake of LS testing guidelines has been low.^28^ The ‘Time to Test’ report published by Bowel Cancer UK in 2018 demonstrated that NICE guideline DG27 was not implemented and recommended that healthcare providers work together to address this underperformance for the benefit of people with LS.^29^ Furthermore, data from National Disease Registration Service (NDRS), indicates that 1.3% of patients with CRC/EC were offered germline genetic testing for LS in 2019, whereas we would estimate that approximately 6%–7% of patients should be offered testing if NICE guidelines were implemented.^26 27 30 31^

Another approach to deliver effective diagnosis is to develop ‘mainstreaming’ models whereby patients are offered constitutional genetic testing by their cancer treating teams locally, rather than relying on referral of eligible patients to tertiary services such as clinical genetics.^32^ This has many possible advantages including shorter timescale to diagnosis, effective communication provided through an existing relationship between patients and their clinical teams, and ensures that eligible patients access testing.^33 34^ This model is associated with high levels of acceptability for patients and clinicians,^32 34 35^ however, relies on the development of new skills by cancer teams.^36^

In the NHS, cancer multidisciplinary teams (MDTs) direct investigations for new cancer diagnoses (including tumour testing for LS), develop treatment plans and follow patients up through their treatment pathways. Regionally in England, cancer MDTs are supported by 21 CAs who provide logistical support, manage variation and ensure quality of diagnostics and subsequent care delivery.

Genomic testing in the NHS is largely provided by 7 Genomic Laboratory Hubs (GLHs) and initiated by/offered through 17 regional clinical genomics services with leadership provided by 7 NHS Genomic Medical Service Alliances (GMSAs). The GMSAs were established in January 2021 to ensure that genomics was embedded in routine clinical care, and therefore, they have an important role in liaising between cancer teams and genomic medicine providers (https://www.genomicseducation.hee.nhs.uk/wp-content/uploads/2022/08/GMSA-map2-1351x630.png).^18 26–31 37^

### New models of care for the benefit of people with LS in the UK

There are significant opportunities to improve the diagnostic pathway for people with LS. In 2017, an expert group of clinicians, charities and patients developed a consensus statement which recommended:

- A national registry of individuals with LS.
- A quality-assured surveillance programme.
- A dedicated clinical champion for LS within each multidisciplinary cancer team.^38^

Since this consensus meeting, NHS cancer and genomics teams have engaged with expert advisors to develop improved pathways for patients. A NICE quality standard was subsequently published which recommends that there is ‘evidence of local arrangements to ensure that there is a clinical lead responsible for implementing the testing pathway for LS’.^39^ Additionally, the NHS England cancer team released a handbook for CAs which recommended that cancer MDTs take responsibility for initiating and completing LS testing pathways in CRC and EC patients via mainstreaming and liaison with regional expert centres.^40^

An optimal framework for lifelong LS care had to demonstrate a survival benefit while simultaneously reducing the costs to the NHS. Therefore, stakeholders were asked to establish goals by which the service could be evaluated. After consideration, the following goals were agreed:

- Ascertainment of new diagnoses of LS through mainstreaming, and cascade testing of at-risk relatives.
- Development of a national patient registry linked to surveillance and other lifelong interventions.
- Measurement and performance management of geographical variation in diagnosis and subsequent management of people with LS.
- A national tiered LS network of cancer teams linked to regional multidisciplinary expert centres with formal structures and referral pathways, which includes MDT discussion on complex cases, monitoring and governance of diagnostic and treatment pathways.
- Regional expert multidisciplinary teams which include gastroenterologists, surgeons, oncologists, specialist nurses who work in tandem with genetic counsellors, geneticists and GLHs/GMSAs.
- Training for providers linked to these networks with a focus on clinical leadership and upskilling of oncologists so as to deliver the relevant personalised therapies.
- Support from NHSE cancer and genomics teams to link CAs with GMSAs and GLHs/pathology networks in order to provide a structure for this care.
- Application of this structure for innovation and translational clinical research with the aim of improving patient care.
- Patient and charity involvement in service development and delivery.

The development of robust improvements to patient care which are meaningful to patients would require a multiprofessional collaborative effort across our geographies. This would only be effectively achieved by building expertise within cancer teams and testing the system through a pilot study before wider buy in could be guaranteed. Therefore, a region was selected that was supported and coached by a network expert MDT and genomics centres as part of a formal disease focused network.

### Pilot project

Royal Marsden Partners CA launched a quality improvement project (QIP) in 2019 to implement effective diagnostic pathways for LS in CRC, which links nine CRC MDTs across the two GMSAs in the West London CA (led by KM).^41^ The goals of this programme were to reduce variability in access to testing pathways, facilitate access to personalised therapy for people with cancer, and strengthen links between local cancer MDTs and regional expert centres within GMSAs. LS champions were appointed in each CRC MDT to endure clear responsibility for testing delivery.

In addition, a core educational component was developed linked to mainstreaming of genomic testing by members of CRC MDTs, with safety netting of patient outcomes by regional genetics services. The establishment of formalised networks linking cancer MDTs to regional expert centres has enhanced communication, training opportunities and standardisation of care, with embedded genomics leads who have responsibility for local delivery of testing and care pathways. The number of patients eligible for constitutional (germline) testing for LS who were identified and effectively referred increased from 10% to 74% across the life cycle of this QIP.^42^ These data formed the basis for a successful bid to NHS England to fund a national transformational project.

### The GMSA LS transformation project

The GMSA National LS Transformation Project led by North Thames and South East GMSAs aims to establish robust testing pathways delivered by cancer teams with a ‘bottom-up’ approach whereby responsibility for testing is clear, and supported by a national infrastructure. This testing should ensure equity of access to diagnosis and linked lifelong care for people with LS. The optimal testing pathway should include delivery of genetic testing by cancer teams locally (‘mainstreaming’).

### Aims of the LS national transformation project

- Providing national and regional medical and nursing leadership and expertise to drive awareness, provide training, facilitate pathway improvements and support the reduction in testing variation and overall compliance with testing guidelines.
- Delivering a comprehensive awareness and upskilling programme.
- Funding NDRS to develop national data solutions to support performance monitoring and improvement along the full Lynch testing pathway for all patients diagnosed with CRC or EC.
- Supporting local audit programmes to inform testing rates, identify and address barriers to reflex Lynch screening and onward referral to Clinical Genetics Services for germline testing, or germline testing within a mainstreamed model.
- Supporting the creation and maintenance of a national Lynch registry and access to the national Bowel Screening Programme for LS patients.
- The introduction and evaluation of mainstreamed pathways where germline testing is provided by cancer teams.
- The implementation of regional expert networks to support mainstreamed pathways.

### Project initiation

A national team led by two clinical leads (KM and ACS) which includes a national LS nurse and two project managers was formed (table 1). Each GMSA appointed a clinical lead and an LS nurse to oversee training within their geography. The national team currently arrange monthly national oversight group meetings which includes representation from 21 CAs and 7 GMSAs, in addition to representatives from cancer and genomics teams at NHSE. The oversight group also includes membership from specialist CRC and EC experts and NDRS.

**Table 1:** The national, regional and local project team

Team member	Responsibility
Lynch champion	Coordination of testing pathway, identifying roles and responsibilities of colleagues within local cancer team, planning development of optimal ‘mainstreaming’ model.
Cancer team	Measurement of performance by completion of local audit tool. Delivery of testing with individual roles linked to guidance developed by NHS England in LS handbook ‘Implementing Lynch syndrome testing and surveillance pathways’ https://www.england.nhs.uk/wp-content/uploads/2021/07/B0622-implementing-lynch-syndrome-testing-and-surveillance-pathways-may-2023.pdf
Regional clinical lead	Supporting delivery locally by cancer teams by identifying areas of good practice, specific issues relevant to local teams by reviewing audit data and arranging one-to-one team meetings to develop action plans. Running workshops and open forums to educate and provide practical support.
Regional LS nurse	Providing peer-support to nursing and medical workforce regionally, including delivery of on-site workshops at each individual cancer team.
Regional project team	Cancer alliances and GMSA alliance project managers: Appointment of LS champions, supporting local stakeholders to ensure delivery of testing.
National team	Coordination of national strategy, ensuring standardisation of approach and equity of access. Running national workshops, developing online training modules. Measuring and reporting performance.

GMSA, genomics medical service alliance; LS, Lynch syndrome; NHS, National Health Service.

#### Patient and public involvement

Patient representatives were appointed to the oversight group including those representing the charities LS UK, Bowel Cancer UK and the Eve Appeal. Patients with LS participated in working groups to share their personal journeys as well as sharing feedback from other patients that illustrated their challenges and outlined the importance of care coordination to relevant stakeholders and contribute to the development of patient information. The patient charity representatives disseminated project communication to their membership and supporters.

The first meeting of the oversight group was held in May 2021 at the outset of the project. CAs were asked to identify an LS champion within each cancer team. Baseline key performance indicators (KPIs) for year 1 of the project were agreed among the oversight group. These KPIs included;

- A 50% increase on testing across each step of the testing pathway (MMR tumour testing, methylation or *BRAF* testing, and constitutional genetic testing) compared with baseline level measured by NDRS.
- Appointment of an LS champion in each CRC and EC MDT.
- Completion of a qualitative baseline survey of perceptions of testing level, barriers and solutions by each champion.
- Development of standardisation of reporting for pathology.
- Development of a rapid registration cancer dataset by NDRS (designed to provide a monitoring tool to continuously assess testing performance).
- Identification of all diagnosed LS patients by each GMSA (within their own geography).
- Development of electronic informatics and testing applications through collaboration.
- Development of online training modules and national training workshops for members of the CRC or EC MDTs, primary care and pathologists.

At each monthly national oversight meeting a set of actions was agreed for GMSAs and CAs, respectively.

Data analysis was performed by a project data analyst in coordination with the NDRS. These data included clinicopathological data from all patients with cancer diagnosed across England, including somatic and constitutional testing outcomes. Results indicated that MMR testing was performed in only 44% of all CRC and EC patients in 2019, with 1.3% of CRC/EC patients offered genetic testing (cancerstats.ndrs.nhs.uk, and manuscript submitted), well below the target threshold of 6%–7%, and below perceived testing levels indicated in the survey of LS champions described below. Subgroup projects were also established in nursing, pathology, the extension of testing to non-CRC non-EC and primary care.

### Appointment and baseline survey of cancer MDT LS champions

**Figure 1:**
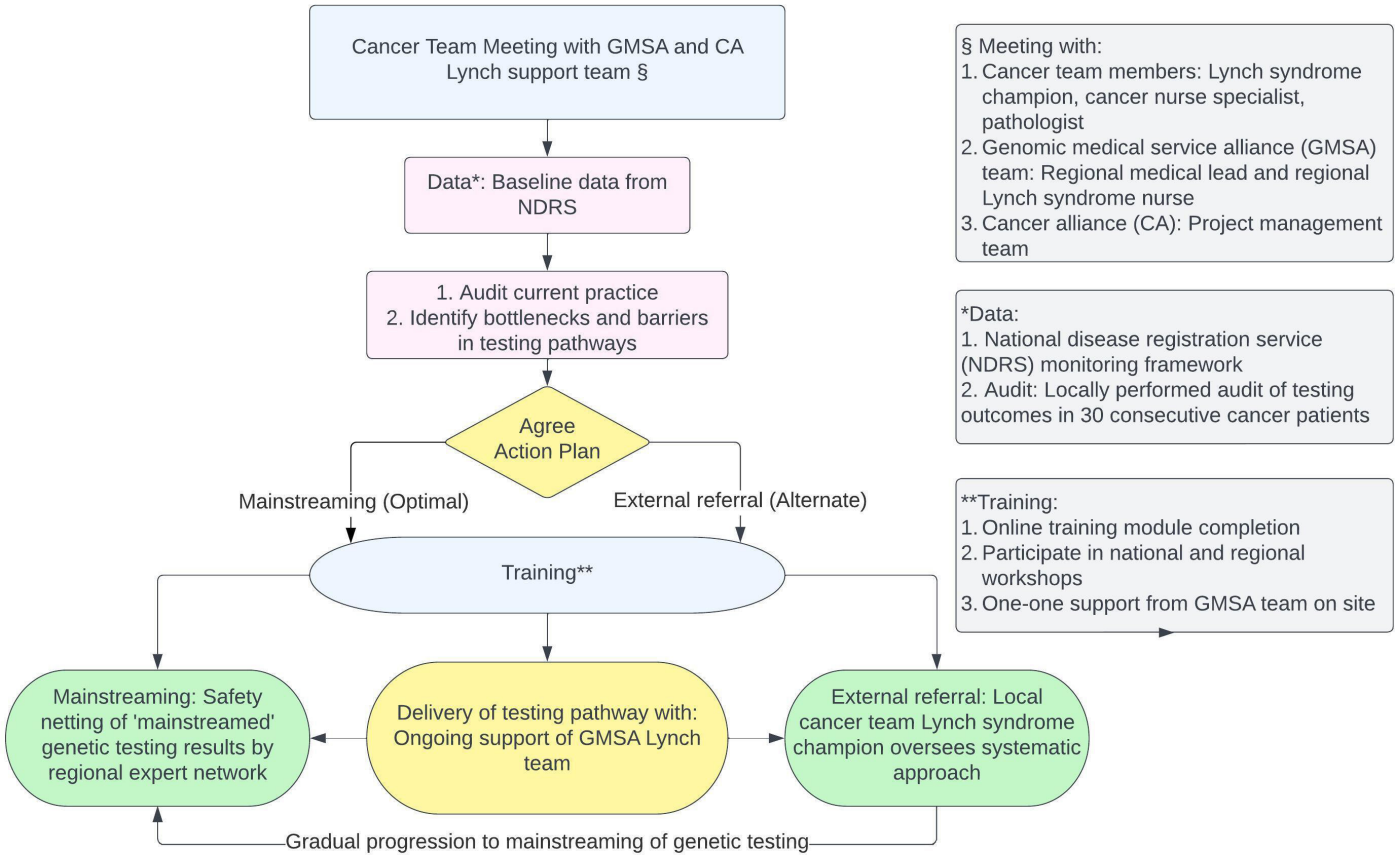
Flow chart for support of delivery of LS diagnosis by cancer teams (details of this pathway are outlined in the section ‘effective diagnostic delivery with mainstreaming’). LS, Lynch syndrome.

In order to deliver testing, each cancer MDT was asked to identify a responsible local lead for the Lynch diagnostic pathway (a ‘Lynch champion’ figure 1). The champion was asked to allocate specific responsibilities within their team, ensure there were systems in place to identify patents who are eligible for genetic testing, and that these patients are offered testing. In 2022, NICE recommended that each CRC MDT identifies a lead within each cancer team, with evidence of local arrangements to ensure delivery.^39^

A national survey was performed with representation from LS champions from each cancer team. In total, 126 responses were recorded from cancer MDTs of which 59 were from CRC MDTs and 67 were from EC MDTs (a 70.7% response rate). The majority of respondents were surgeons (74%). Champions were asked about local testing practices as well as barriers and potential solutions to improve delivery of testing according to NICE guidelines.

**Figure 2:**
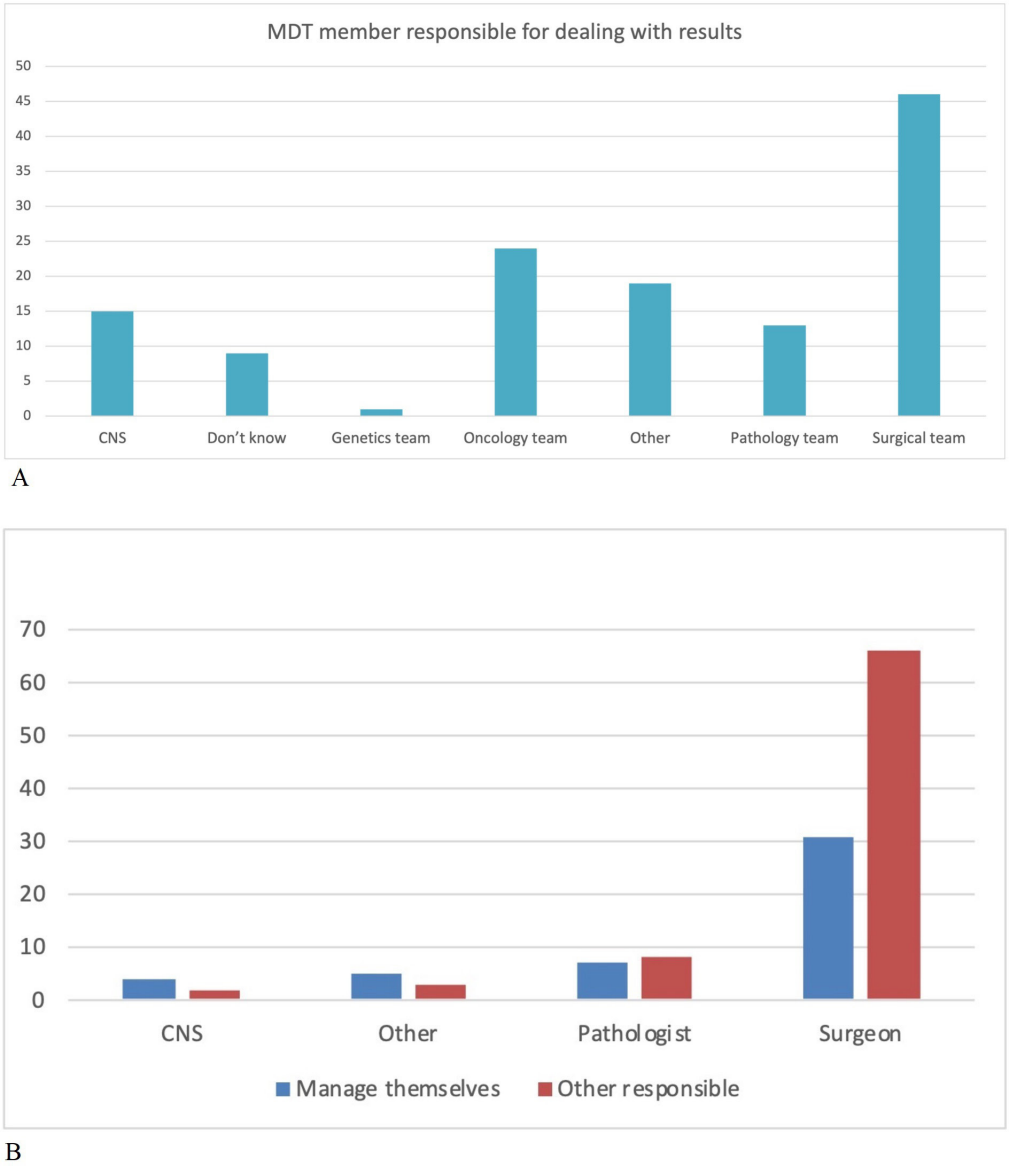
Cancer multidisciplinary team (MDT) member responsible for (A) receiving and actioned somatic testing results (B) Respondents’ specialty (blue columns); and opinion about which specialists they feel should manage these patients (red columns). The majority of respondents felt that someone else should be managing their patients at high risk of hereditary colorectal cancer (from baseline survey of LS champions). Y-axis=numbers of respondents. CNS, cancer nurse specialist; LS, Lynch syndrome.

From the survey, notable variations in practice perceptions were identified. The responsible clinician for actioning genetic referral was variable between MDTs, with 81/126 (64.2%) of respondents believing that the responsibility for following up results was with another member of the team rather than them personally (figure 2). Furthermore, 32/126 (25.4%) were not aware if the index tumour MMR test was IHC or MSI in their institution, and 35 (27.8%) offered both IHC and MSI. Among EC champions, 19/67 used MSI as a first line test, and 22/67 were unsure if they used IHC or MSI, despite only IHC being recommended according to NICE guidelines for use in EC.

**Figure 3:**
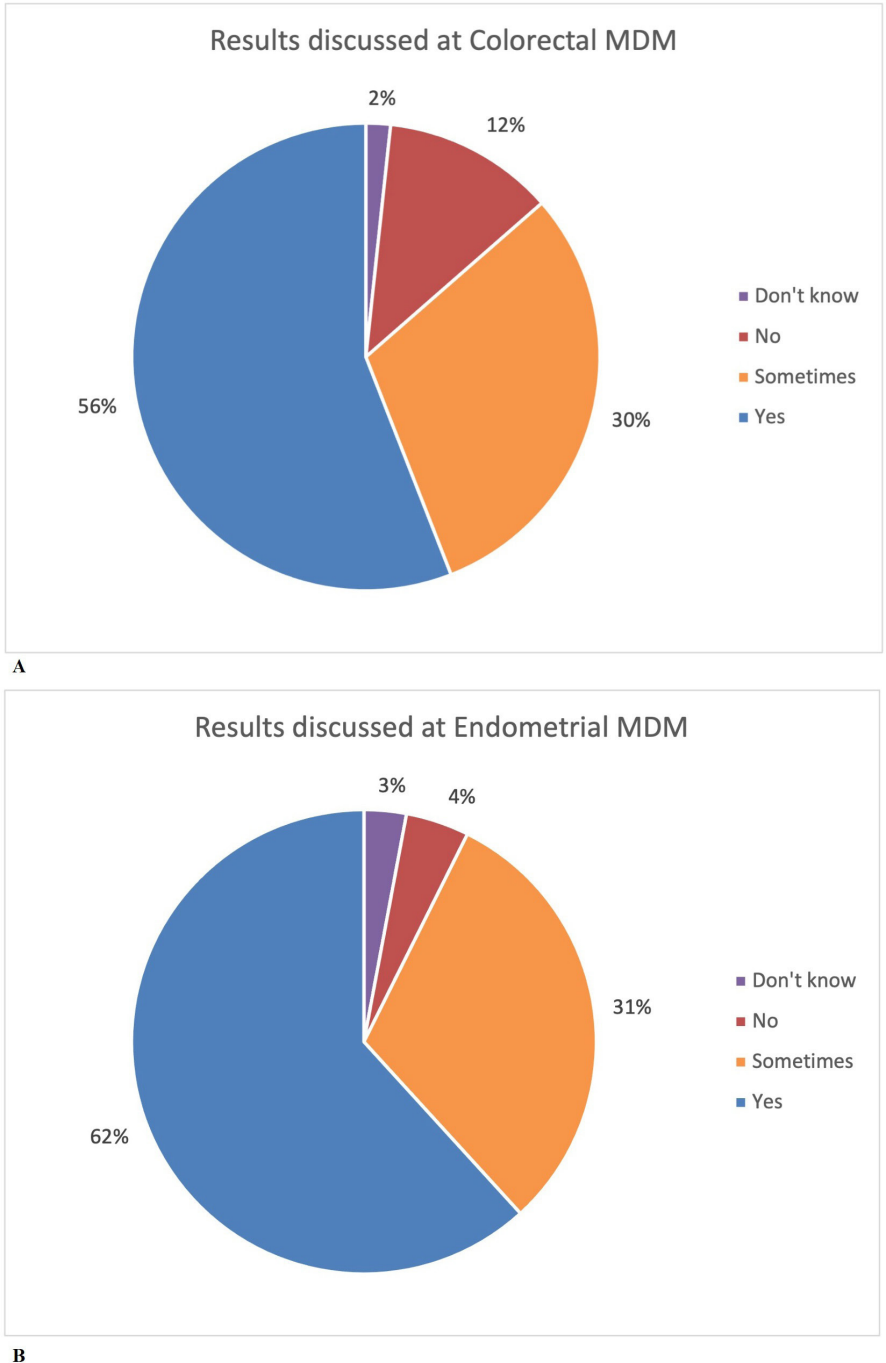
Patient results of MMR testing discussed at weekly MDT meeting (MDM) for colorectal and endometrial cancer (from baseline survey of LS champions). Discussed: yes/no/sometimes/don’t know. LS, Lynch syndrome; MDT, multidisciplinary team; MMR, mismatch repair.

Results of MMR testing were reported to be discussed at 56% of CRC and 62% of EC MDT meetings (figure 3). Respondents were not aware in 61/126 (48.4%) responses if they performed methylation testing where it was indicated, although they perceived that 82/126 (65%) of MDTs pathologists would automatically arrange methylation testing. Additionally, 57/126 (45.2%) stated that patients would be referred to clinical genetics prior to the point of eligibility for genetic testing.

In total, 71% of CRC MDTs and 66% of EC MDTs stated they offered ‘universal testing’ for LS in accordance with NICE guideline recommendations. Of CRC or EC MDTs 17% reported that universal testing is offered for some cases, and the remainder reported that the recommended universal testing is never offered. The perception of ‘universal testing’ by respondents was that it represented tumour MMR testing not including constitutional diagnostic testing for LS however, with only 28% reporting that they offered genetic testing. Referral for genetic testing was often performed in an ad hoc way without a consistent approach (88%). Most cancer MDTs (89%) were not aware of how many of their patients accessed genetic testing. Reassuringly, 32% reported they would like to be ‘upskilled’ to offer genetic testing locally without external referral. A further 15% felt that a reliance on genetics departments for testing led to a pathway and workload that need to be reviewed, or training and support is required before implementation.

**Figure 4:**
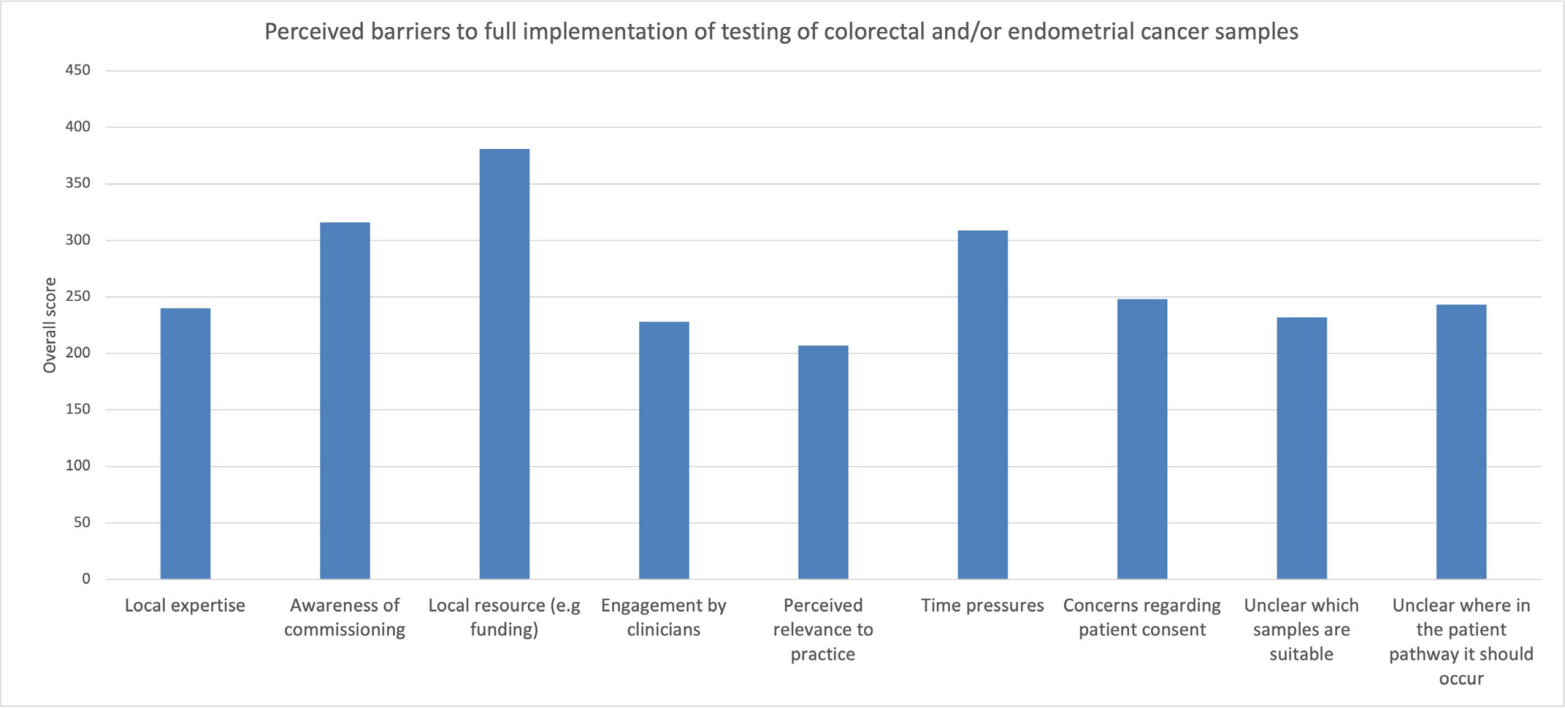
Reported barriers to implementation from baseline survey of LS champions (respondents were asked to complete a Likert scale with higher scores presenting more significant barriers). LS, Lynch syndrome.

A 5-point Likert scale was used to prioritise perceived barriers to testing. The main barriers to delivery were reported to be local resource including funding, awareness of regional service commissioning structures, and time pressure (figure 4). Cancer teams suggested that the solutions to delivery of comprehensive testing could be provided with clearer protocols and pathways, sharing of experiences from other institutions and training.

### Effective diagnostic delivery with mainstreaming: supporting the process

Workforce training was further developed from the baseline training delivered by the RM Partners QIP, focused on overcoming barriers to testing, identification of eligible patients and mainstream constitutional testing.

Training has been developed to support cancer team champions and their colleagues, with online training modules supplemented by workshops delivered by regional LS nurses and GMSA teams which are focused on identification of solutions tailored to specific issues within individual cancer teams.

The online modules are designed to support teams nationally to improve delivery of their local diagnostic pathway for LS, and therefore, improve uptake of genetic testing for LS through integration into standard cancer clinics. There are two stages to this training, first to identify eligible patients for genetic testing, and subsequently to deliver mainstream genetic testing locally with training which includes consent, informing patients of their genetic test results and determining how people should be managed following diagnosis of LS. These training modules are deliberately discrete and therefore designed to be palatable to busy clinicians. Modules have been developed for all members of CRC and EC teams, and additional modules for primary care clinicians and pathologists.

When cancer team clinicians have completed the modules they meet with the GMSA team to discuss local barriers to testing and how they might be mitigated. Cancer teams are asked to complete an audit of 30 consecutive patients with cancer to identify shortcomings in their local testing pathway which might be addressed. It is recommended for example that pathologists ‘reflex’ arrange MMR tumour testing and *MLH1* methylation and/or *BRAF* testing in all eligible patients, but that patient-facing clinicians receive and action the test reports to ensure eligible patients are offered constitutional testing in a systematic fashion. In addition, the roles and responsibilities of individual cancer team members can be clarified and supported by a local LS champion who will have overall responsibility for ensuring a coordinated approach is delivered.

When this pathway is established the GMSA team including LS nurse will arrange workshops with cancer team clinicians who are now ready to provide mainstreamed genetic testing to their patients with cancer. Molecular testing is funded through national commissioning following the eligibility criteria set out in the National Genomic Test Directory. The remainder of the pathway (including IHC) was supported through transformational funding.

### Expert networks for LS

Although most patients from day to day will be managed in primary and secondary care, many patients have complex needs that benefit from a multispecialty and multidisciplinary coordinated approach that is best delivered through a centre of regional expertise.^5 43^ A specialist regional LS network may facilitate mainstreaming, and managing ongoing care, following a diagnosis of LS. These networks will link cancer team clinicians locally with regional experts across multiple specialties including pathology, gastroenterology, genetics, gynaecology and other relevant clinicians.

We recommend a three-tier structure in line with guidance from the Association of Medical Royal Colleges Genomics Professional Partnership Group.^43^

1: A National Centre.

2: Regional Expert Networks (within each GMSA/CA geography).

3: Local leadership within cancer teams (ie, local champions/genomic advisors).

National and regional centres would work together to ensure that variation in access to care is addressed, mainstreaming services are supported, with involvement of representatives of the professionals who provide clinical care for people with LS. The purpose of a national centre is to provide strategic advice, support and identify variation between regional expert networks on behalf of NHSE.

### Regional network activities

Optimal management of LS syndrome may include a regional expert clinical team aligned GMSAs and GLHs and linked to cancer MDT LS champions. The regional specialist team would offer advice and support to local MDTs and have a role in the development of national services. The network will ensure and monitor equity of access for patients with LS, support mainstreaming pathways, and help manage their lifelong care.^44^

Additionally, expert centres and regional networks can offer specialist MDT meetings and/or ‘virtual review’ of patients from other centres who will receive management locally but for whom support may be given in management decisions and/or specific treatments, for example, segmental or extended resection in LS, resection of CRC in FAP patients, advise about the appropriateness of potential referrals to expert centres, and decision-making around prophylactic gynaecological surgery.

### A national registry of LS

The GMSA project asked each regional genetics service to identify every patient diagnosed through their service since diagnostic testing for LS commenced in the 1990s, and to maintain a prospective registry. Complete ascertainment of all genetically confirmed cases by GMSAs was requested using an agreed standard dataset including evidence of a pathogenic or likely pathogenic constitutional variant in an MMR gene, in order to ensure that people with LS are able to access improvements to their care and maximise the benefit of interventions designed to mitigate their risk of cancer.

The information about ascertained LS patients was shared with NDRS to facilitate recruitment to national screening programme following the announcement by NHS England in December 2021 that people with LS would undergo colonoscopic surveillance via the bowel cancer screening service from mid-2023. The goal of colonoscopic surveillance delivered in this way would be to ensure high quality colonoscopy, timely call and recall, and improvements in patient experience. Colonoscopy will be delivered in line with UK guidelines for the management of people with LS.^1 5^ In total, just over 9000 people with LS were identified via regional genetics services, which is consistent with the estimation that only 5% of all diagnosed of LS have been made in the whole population in England. Selected from this cohort just under 7000 people with LS are currently age eligible for colonoscopic surveillance and will be invited using the national screening mechanism.

## Conclusions and future directions

There are limitations of a ‘bottom-up’ approach with mainstreaming of constitutional testing by cancer treating clinical teams. The scale of the problem is a significant challenge which requires matched resource to train clinical staff, identify specific bottlenecks in local services, manage variation and ensure sustainability. The workforce among cancer teams have had relatively little previous experience of genomics in clinical practice, which underlines the novelty and challenge of this approach. The use of audit is predominantly to identify specific issues which may be addressed locally but is not a sustainable model for national quality improvement. Therefore, measurement of variation in performance requires a nationally coordinated data solution which identifies patient populations who are not accessing diagnostic testing, and which facilitates support for the relevant clinical teams in those regions and is under development by NDRS.

Additionally, the testing pathway as defined by existing NICE guidelines is a multistep pathway, with degradation of testing as each step represents a bottleneck. The likely evolution of testing to a single-step paired somatic-germline testing pathway is imminent and likely to circumvent many of these barriers to testing. However, by training a workforce and identifying clear lines of responsibility for testing, delivered by local cancer teams and led by local LS champions, new testing algorithms may be more effectively implemented. Embedding LS testing requires models of sustainability which will need to ensure testing continues beyond the lifespan of this national project which completes in April 2024. Given the lag period between cancer diagnosis and genetic diagnosis of LS the longer-term project impact will be measurable in 2026 through NDRS reporting.

Significant improvements in care are feasible to ensure effective lifelong management of people with LS. Increased awareness of cancer risks and interventions to manage these risks have facilitated the recent evolution of care in the UK, however, delivery of diagnosis is required to leverage these opportunities to improve outcomes for patients, for example, with the introduction of a national quality assured colonoscopic surveillance programme in England which launched in July 2023. Through the English LS transformation project, we have further developed expertise in this condition within cancer treating teams. This is aligned to leadership and clearly defined team roles designed to deliver diagnosis of LS after a diagnosis of cancer, with patients therefore having improved access to precision medicine treatment and effective management of cancer risk in their families.

Responsibility for the provision genetic cascade testing remains with specialist genomics services. Nevertheless the lifelong care of people diagnosed with this condition depends on diagnosis and awareness of the population with LS. This population has been more clearly defined through a national LS registry developed in coordination with NDRS. Improved infrastructure with the development of regional multidisciplinary expert networks will continue to provide sustained support to mainstreaming clinicians and multidisciplinary management of complexity in clinical care. We believe that the NHS, because of equity, standardisation of care and universal access, can deliver these benefits however this work will require reinforcement from multiple sources including GMSAs and CAs.

The English National LS Transformation Project continues to evolve and embed genomics throughout cancer MDTs in England, with the establishment of new mainstreaming teams, improved access to diagnostic testing for LS in patients with cancer and delivery of NICE guidelines DG27 and DG42. The long-term outcomes of this project will be reported in future publications.

## Data Availability

Data are available on reasonable request. Data are available for NHS staff.
